# MS/MS-Guided Isolation of Clarinoside, a New Anti-Inflammatory Pentalogin Derivative

**DOI:** 10.3390/molecules23051237

**Published:** 2018-05-22

**Authors:** Coralie Audoin, Adam Zampalégré, Natacha Blanchet, Alexandre Giuliani, Emmanuel Roulland, Olivier Laprévote, Grégory Genta-Jouve

**Affiliations:** 1 Laboratoires Clarins, 5 rue Ampère, 95300 Pontoise, France; coralie.audoin@clarins.com (C.A.); adam.zampalegre@clarins.com (A.Z.); natacha.blanchet@clarins.com (N.B.); 2 DISCO Beamline, Synchrotron SOLEIL, 91192 Gif-sur-Yvette, France; alexandre.giuliani@synchrotron-soleil.fr; 3 UAR1008, CEPIA, INRA, 44316 Nantes, France; 4 C-TAC, UMR 8638 CNRS, Faculté de Pharmacie de Paris, Université Paris Descartes, Sorbonne Paris Cité, 4 Avenue de l’Observatoire, 75006 Paris, France; emmanuel.roulland@parisdescartes.fr (E.R.); olivier.laprevote@parisdescartes.fr (O.L.); 5 Department of Biochemistry, Hôpital Européen Georges Pompidou, AH-HP, 75015 Paris, France

**Keywords:** *Mitracarpus scaber* Zucc., pentalogin, anti-inflammatory, MS/MS, Il-8

## Abstract

Re-investigation of the chemical composition of the annual plant *Mitracarpus scaber* Zucc. led to the identification of clarinoside, a new pentalogin derivative containing a rare quinovose moiety, and the known compound harounoside. While the planar structure was fully determined using tandem mass spectrometry (MS) and quantum mechanics (QM) calculations, the tridimensional structure was unravelled after isolation and NMR analysis. The absolute configuration was assigned by comparison of experimental and theoretical synchrotron radiation circular dichroism spectra. Both compounds were tested for anti-inflammatory activity, and compound **1** showed the ability to inhibit the production of interleukin-8 (Il-8) with an IC${}_{50}$ value of 9.17 μM.

## 1. Introduction

**Figure 1:**
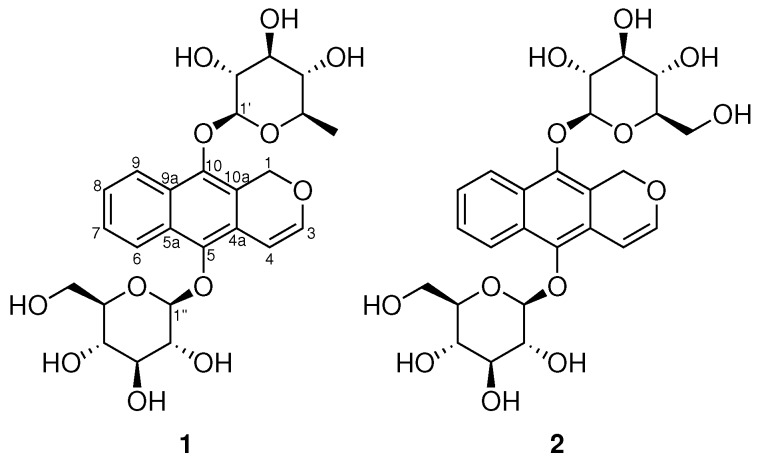
Structures of clarinoside (**1**) and harounoside (**2**).

Mass spectrometry (MS) has become a very convenient technique for the targeted search of new bioactive metabolites [1,2], and the recent introduction of the Global Natural Product Social Molecular Networking (GNPS) Web platform (http://gnps.ucsd.edu) has enabled the quick and automatic spectral mining of MS/MS spectra [3]. In our ongoing research for bioactive compounds, we decided to re-investigate the chemical composition of *Mitracarpus scaber* Zucc. using a MS/MS-guided approach. *M. scaber* is an annual plant used in African traditional medicine endowed with antifungal, antimicrobial and anti-inflammatory properties [4,5]. Indeed, in West Africa, the leaves of *M. scaber* are widely used for headache, toothache, amenorrhea, dyspepsia, hepatic diseases, venereal diseases, leprosy, and for the treatment of skin diseases such as scabies, infectious dermatitis, and eczema. It is well known to contain phenols [5], flavonoid glycosides [5], furanocoumarines [5], terpenes [6], alkaloids [7], and pentalongin derivatives [8,9]. Herein, we report the identification of clarinoside (**1**), a new pentalongin derivative exhibiting the rare quinovose moiety along with the known harounoside (**2**) (Figure 1). Both compounds were tested for anti-inflammatory activity by evaluating their ability to inhibit the production of interleukin-8 (Il-8).

## 2. Results

**Figure 2:**
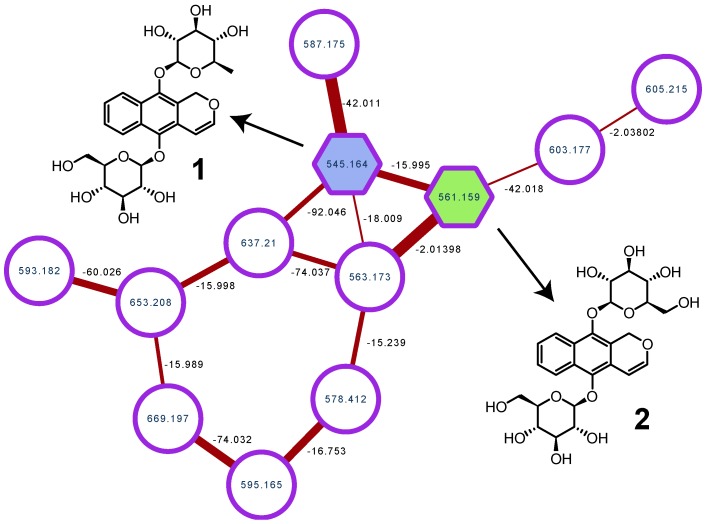
Selected cluster containing clarinoside (**1**) and harounoside (**2**).

The analysis started with the creation of a molecular network of the ethanolic extract of *M. scaber*. The data-dependent analysis (DDA) LC-MS/MS data were uploaded to the GNPS platform, and a network was generated using the parameters listed in the Materials and Method section below (Figure 2). As an anchor (reference) compound, harounoside (**2**) was used. Its node was quickly “illuminated” using the high-resolution MS data and fragmentation pattern. The *m*/*z* value at 561.159 corresponding to the [M + Na]${}^{+}$ adduct of **2** was identified, and two diagnostic MS/MS fragments were present on the spectrum (see Supplementary Materials): the first resulting from the cleavage of the glycosidic bond between the aglycone and a glucose moiety at *m*/*z* 399.1080 [M-Glc + Na]${}^{+}$, and the second at *m*/*z* 236.0452 resulting from the cleavage of the second O–C between the aglycone and the other glucose [M-2Glc + Na]${}^{•+}$. In order to find the structurally related compounds, the cluster was further studied by annotating the edges with *m*/*z* differences corresponding to known (bio)chemical modifications implemented in the MetaNetter 2 package [10]. Out of the 307 nodes of the network, 1 node directly connected to harounoside (**2**), with a *m*/*z* difference of −15.995 attracting our attention. According to the biotransformation list available in the MetaNetter package, this difference corresponded to a dehydroxylation.

**Figure 3:**
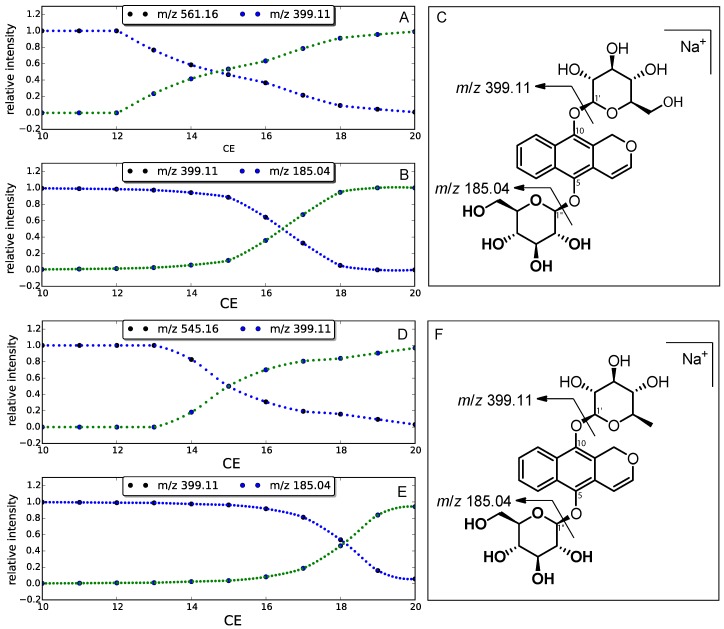
Plot of relative ion current vs collision energy corresponding to *m*/*z* 561.16 vs. 399.10 (**A**) and *m*/*z* 399.10 vs. 185.04 (**B**). (**C**) MS/MS fragments of **2**. Plot of relative ion current vs. collision energy corresponding to *m*/*z* 545.16 vs. 399.10 (**D**) and *m*/*z* 399.10 vs. 185.04 (**E**). (**F**) MS/MS fragments of **1**.

Considering the structure of **2**, dehydroxylation could occur at several positions of each of the two sugars; in order to know on which side of the compound **2** the dehydroxylation site was located, the fragmentation of both glucose moieties was studied using quantum mechanics (QM). After examination of the MS/MS spectrum, the sequential losses of two glucoses were observed. The nature of the sugar being the same at C-5 and C-10, the energy level of the two O–C bonds was only related to the position on the aglycone. In order to confirm this hypothesis, the energy profile of the homolytic dissociation was predicted using the B3LYP method at the STO-3G level (see Supplementary Materials). The calculations predicted a difference of ca. +2.5 eV in favor of the O–C1${}^{\prime \prime }$, indicating that the first fragment observed at *m*/*z* 399.1080 was related to the loss of one glucose at C-10. This energy difference was very supportive; on the basis of these theoretical results, an energy-resolved mass spectrometry (ERMS) study [11] was undertaken in order to determine the stability of the two O–C bonds (O–C1${}^{\prime \prime }$ and O–C1${}^{\prime }$) of compound **2**. After selection of the parent ion at *m*/*z* 561.16, the intensity of the ion at *m*/*z* 399.11 was recorded using an increasing value of collision energy (). After the complete extinction of the parent ion (Figure 3A), the daughter ion at *m*/*z* 399.11 was then fragmented into one major ion at *m*/*z* 185.04 using the same approach (Figure 3B). As shown in Figure 3, the O–C1${}^{\prime }$ bond linking the aglycone to the glucose moiety was weaker than the O–C1${}^{\prime \prime }$ bond, as it required a lower collision energy value for a 50% dissociation (ca. 15 and 17 for O–C1${}^{\prime }$ and O–C1${}^{\prime \prime }$, respectively).

The experimental data confirmed the QM predicted values, and the same ERMS approach was used for compound **1**. As observed in Figure 3D,E, an increase in the collision energy value was required in order to produce the ion at *m*/*z* 185.05 (ca. 15 and 18 for O–C1${}^{\prime }$ and O–C1${}^{\prime \prime }$, respectively). These results clearly confirmed the position of the deoxyhexose moiety at C-10. The planar structure was further confirmed by the identification of the neutral loss of 146.0605 Da resulting from the difference between the [M + Na]${}^{+}$ ion at *m*/*z* 545.1685 and the fragment at *m*/*z* 399.1080. In parallel with the loss of 162.05 Da corresponding to a glucose moiety (observed for **2**), the loss of 146.0605 was consistent with a deoxyhexose such as fucose, rhamnose, or quinovose. Unfortunately, and despite the use of a recent methodology to distinguish the mono-saccharides using MS/MS [12], it was not possible to determine the relative stereochemistry of the sugar moieties using MS analysis only; thus the isolation of compound **1** was undertaken.

After a reverse-phase high-performance liquid chromatography (HPLC) purification, 84 mg of compound **1** was obtained, and a full set of NMR experiments was performed. The structure of the aglycone was confirmed by comparison of the ${}^{1}$H and ${}^{13}$C NMR chemical shifts (see Table 1). The nature of the sugar was determined by taking advantage of the newly published methodology by Giner et al. [13], which is based on the acid-promoted hydrolysis of the studied compound performed directly in the deuterated NMR solvent. Looking at the ${}^{1}$H NMR spectrum, the doublets at δ 4.66 (*J* = 7.7 Hz) and 4.79 (*J* = 7.8 Hz) ppm clearly confirmed a glucose and a quinovose moiety (see Supplementary Materials). According to the ERMS data, the quinovose was located at C-10, and this was confirmed by the two ${}^{3}$*J* coupling between H-1${}^{\prime }$/C-10 and H-1${}^{\prime \prime }$/C-5 on the HMBC spectrum. The detailed NMR data are given in the Table 1.

**Figure 4:**
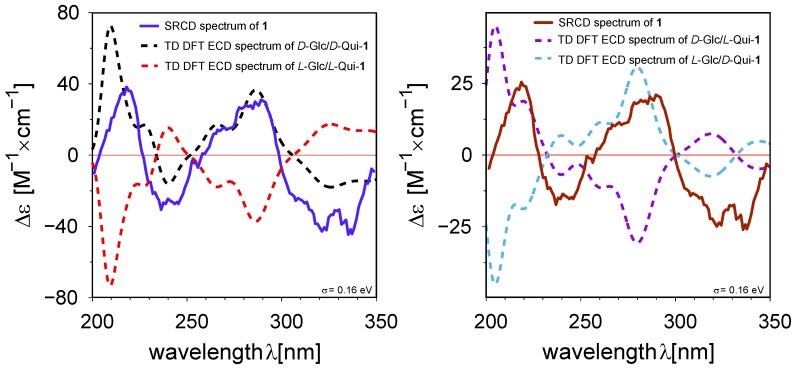
Overlay of synchrotron radiation circular dichroism (SRCD) and TD DFT spectra of **1**.

The absolute configuration of compound **1** was determined by comparison of a synchrotron radiation circular dichroism (SRCD) spectrum with a time-dependent density functional theory (TD DFT) theoretical electronic circular dichroism (ECD) spectrum (Figure 4). Unexpectedly, the ECD spectrum was quite complex with four Cotton effects of alternative signs. The calculations were run on the four diasteroisomers, that is, d-Glc/d-Qui, d-Glc/l-Qui, l-Glc/d-Qui, and l-Glc/l-Qui. While the absolute d configuration of the glucose moiety was expected, as it is well known that higher plants produce only this enantiomer [14], the absolute configuration of the quinovose moiety was not obvious because the quinovose can originate from either d-glucose [15] or l-fucose [16].

A very good agreement was observed between the d-Glc/d-Qui theoretical and the experimental spectra (Figure 4). Compound **1** could be named as 5,10-dihydroxy-2*H*-naphtho[2,3-b] -pyran-5-β-d-glucopyranosyl-10-β-d-quinovopyranoside.

**Figure 5:**
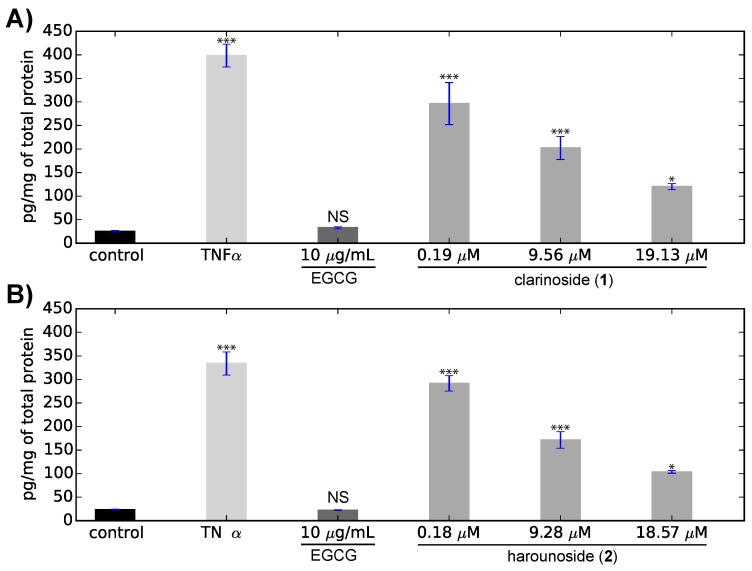
Inhibition of the interleukin-8 (Il-8) production: (**A**) clarinoside (**1**); (**B**) harounoside (**2**). Means ± SD are shown. NS: not significant; * p<0.05; *** p<0.001.

Both compounds **1** and **2** were tested for anti-inflammatory activity by measuring their ability to inhibit the production of Il-8, one of the key mediators associated with inflammation [17,18]. After exposure to the tumor necrosis factor alpha (TNF-α) at 0.5 ng·mL${}^{-1}$ for 24 h, the production of Il-8 was measured and compared to the known anti-inflammatory standard epigallocatechin gallate (EGCG). As shown in Figure 5, the TNF-α induced the production of Il-8 of 398.37 ± 24.09 pg/mg of total protein, while the addition of EGCG at 21.8 μM allowed a return to the basal threshold of 33.01 ± 2.12 pg/mg of total protein.

Although the two compounds were tested during two independent tests, the differences in the measured concentrations (i.e., the production of Il-8 and its inhibition) were observed in both experiments. A good correlation ($R^{2}$ = 0.996) was observed between the inhibition of the production of Il-8 and the concentration of clarinoside (**1**). An IC${}_{50}$ value of 9.17 μM was measured, and a total inhibition of the Il-8 production at 36 μM could be extrapolated. The IC${}_{50}$ value of **1** was of the same order of magnitude as that of EGCG (10.9 μM [19]), although it was slightly lower. Interestingly, the IC${}_{50}$ value of **2** (9.21 μM) was very similar to that measured for compound **1**, indicating that the structural modification had no impact on its biological activity.

To conclude, this study enabled the rapid identification of one new compound from *M. scaber*. The biological activity evaluation highlighted the ability of compounds **1** and **2** to inhibit the production of Il-8, confirming the importance of *M. scaber* metabolites and their possible uses in cosmetics and personal care products.

## 3. Materials and Methods

### 3.1. General Procedure

The preparative HPLC was performed on a VWR LaPrep P110 system using a C-630 Büchi UV detector. NMR spectra acquisition was realized using a 600 MHz Bruker Avance spectrometer equipped with Z-gradients and a triple resonance TXI probe. The signals were referenced in ppm to the residual solvent signals (CD${}_{3}$OD, at $\delta_{H}$ 3.31 and $\delta_{C}$ 49.0). The infrared spectrum was acquired on a Nicolet IS50 FT-IR spectrophotometer. The specific rotation was measured using an Anton Paar MCP150 polarimeter.

### 3.2. Plant Material

The flowered aerial parts of *M. scaber* were collected in Burkina Faso in the town of Poun and then dried in the same area.

### 3.3. Extraction and Purification

An ethanolic extraction was performed on a 300 g sample of the dried plant with a plant/solvent ratio of 1/7 yielding 16.5 g of crude extract, which was then directly processed by reverse-phase HPLC with an XBridge Prep C18, 5 μm (OBD 30 × 250 mm) preparative HPLC column. A gradient H${}_{2}$O/MeOH (from 90/10 to 70/30 in 30 min at 100 mL·min${}^{-1}$) was used to afford compounds **1** (84 mg) and **2** (113 mg).

### 3.4. LC-MS Data Acquisition and Processing

An XEVO-G2 XS QTOF (Waters) equipped with an electrospray ionization (ESI) source was used for the qualitative analysis of the extract. A first screening analysis was performed using the MS${}^{E}$ technology (Waters) on a mass range from 50 to 1500 Da. The optimal ionization-source working parameters were as follows: capillary voltage of 3.0 kV; sampling cone of 40 V; extraction cone of 6.0 V; source temperature of 150 ${}^{\circ }$C; desolvation temperature of 600 ${}^{\circ }$C; cone gas flow of 50 L/h; desolvation gas flow of 1000 L/h. MS/MS data were obtained using a DDA with the same ionization parameters as above using three different collision energies: 10, 20, and 40 V.

### 3.5. Construction of the Molecular Network

A molecular network was created using the online workflow at GNPS [3]. The data were filtered by removing all MS/MS peaks within ±17 Da of the precursor *m*/*z*. MS/MS spectra were window filtered by choosing only the top six peaks in the ±50 Da window throughout the spectrum. The data were then clustered with MS Cluster with a parent mass tolerance of 2.0 Da and a MS/MS fragment ion tolerance of 0.5 Da to create consensus spectra. Furthermore, consensus spectra that contained fewer than two spectra were discarded. A network was then created in which edges were filtered to have a cosine score of above 0.7 and more than three matched peaks. Furthermore, edges between two nodes were kept in the network if and only if each of the nodes appeared in each other’s respective top 10 most similar nodes. The spectra in the network were then searched against GNPS’s spectral libraries. The library spectra were filtered in the same manner as the input data. All matches kept between network spectra and library spectra were required to have a score of above 0.7 and at least six matched peaks.

### 3.6. Energy-Resolved Mass Spectrometry

The LTQ-Orbitrap XL mass spectrometer (Thermo Scientific (Bremen), Bremen, Germany) was used for the ERMS study. The analysis was performed in positive-ion mode with a mass range of *m*/*z* 100–1100. The optimized ESI parameters were set as follows: capillary temperature of 250 ${}^{\circ }$C; sheath gas (nitrogen) flow of 30 arb.; auxiliary gas (nitrogen) flow of 10 arb.; source voltage of 4.25 kV; capillary voltage of 25 V; tube lens voltage of 110 V. The resolution of the Orbitrap mass analyzer was set at 30,000. The isolation width was 2 amu, and the normalized collision energy (CE) was set from 10 to 20. Collision-induced dissociation (CID) was conducted in LTQ with an activation *q* value of 0.25 and activation time of 30 ms. All instruments were controlled by the Xcalibur data system, and the data acquisition was carried out by analyst software Xcalibur (version 2.1) (Waltham, MA, USA) from Thermo Electron Corp.

### 3.7. Synchrotron Radiation Circular Dichroism

The SRCD experiments were carried out on the SRCD station [20] DISCO beamline [21] at the SOLEIL synchrotron (Gif-sur-Yvette, France). The samples were placed in calcium fluoride cells of 100 micron optical path lengths and measured at 0.2 mol/L in methanol. (+)-Camphor-10-sulfonic acid (CSA) solution was used to calibrate the SRCD signal. For each sample, three spectra were collected in the 350–200 nm range with a 1 nm step and 1200 ms integration time. The molar circular dichroism Δϵ is expressed in M${}^{-1}$ cm${}^{-1}$.

### 3.8. Computational Details

All QM calculations were carried out using Gaussian 16 [22]. The energy scan of the C–O bonds was performed using the Hartree–Fock method at the STO-3G level and a 0.1 Å bond-length step. The GMMX package was used for the conformational analysis (force field: MMFF94). The TD DFT calculations were performed using the B3LYP method at the 6-31G(d) level for 20 excited states. SpecDis 1.71 software was used to plot the ECD spectrum [23].

### 3.9. Cell Culture

HaCaT keratinocyte cells were cultured under standard conditions in DMEM supplemented with 10% fetal calf serum. The medium was changed every second day. Confluent cultures were removed by trypsin incubation, and then the cells were counted. They were seeded into 96-well culture microplates at a density of 30,000 cells per well (200 μL) and kept at 37 ${}^{\circ }$C for 24 h.

### 3.10. Interleukine Release Measurement

The release of Il-8 in cell supernatants was determined by ELISA. After TNF-α incubation (0.5 ng/mL), cell supernatants were harvested and stored at −20 ${}^{\circ }$C until use for measurements. The quantity of released Il-8 was measured according to the manufacturer’s instructions (Kit ELISA Human CXCL8 / IL8 R&D Systems). The decrease in Il-8 production by EGCG (10 μg/mL) validated the method.

### 3.11. Statistical Analyses

All statistical analyses were performed using R 3.5.0 [24]. Cell samples were analyzed by repeated measures (n = 4) one-way analysis of variance (ANOVA) followed by a Tukey’s range test. Significant differences for both clarinoside (**1**) and harounoside (**2**) were relative to control as indicated (NS: not significant; * p<0.05; *** p<0.001).

### 3.12. Compound Characterization

**1**: White, amorphous solid; ${\left[\alpha \right]}_{D}^{20}$ +12.8 (c 0.1, CH${}_{3}$OH); UV (DAD) $\lambda_{max}$ 223, 245, 284, 346 nm; ${}^{1}$H NMR and ${}^{13}$C NMR data: see Table 1; HRESIMS (+) *m*/*z* 545.1685 [M + Na]${}^{+}$ (545,16295 calcd. for C${}_{25}$H${}_{30}$O12Na, Δ−1.8 ppm).

## Figures and Tables

**Table 1:** ${}^{1}$H and ${}^{13}$C NMR data for **1** at 600 MHz in CD${}_{3}$OD ($\delta_{H}$ in ppm).

Clarinoside (1)						Harounoside (2) [8]		
**No.**	$\delta_{H}$ **(Multiplicity, *J*)**	$\delta_{C}$	**No.**	$\delta_{H}$ **(Multiplicity, *J*)**	$\delta_{C}$	**No.**	$\delta_{H}$	$\delta_{C}$
1	5.29 (dd, 40.7, 13.8 Hz)	65.2	1${}^{\prime }$	4.66 (d, 7.8)	106.2	1	5.39; 5.30	65.4
3	6.67 (dd, 14.4, 5.9 Hz)	147.8	2${}^{\prime }$	3.61 (dd, 9.0, 7.8)	75.9	3	6.68	147.8
4	6.66 (dd 14.4, 5.9 Hz)	102.1	3${}^{\prime }$	3.38 (t, 9.0)	77.7	4	6.64	102.2
4a		121.6	4${}^{\prime }$	3.11 (t, 9.0)	73.5	4a		121.7
5		143.3	5${}^{\prime }$	3.11 (m)	77.8	5		143.4
5a		131.0	6${}^{\prime }$	1.21 (d, 5.4)	18.1	5a		131.0
6	8.43 (d, 8.2 Hz)	124.7	1${}^{\prime \prime }$	4.79 (d, 7.8)	106.9	6	8.42	124.7
7	7.44 (dt, 8.2, 1 Hz)	127.0	2${}^{\prime \prime }$	3.65 (m)	75.8	7	7.43	127.0
8	7.40 (dt, 8.2, 1 Hz)	126.2	3${}^{\prime \prime }$	3.46 (m)	71.5	8	7.39	126.3
9	8.41 (d, 8.2 Hz)	123.7	4${}^{\prime \prime }$	3.14 (m)	76.9	9	8.43	123.7
9a		129.1	5${}^{\prime \prime }$	3.47 (t, 9.0)	78.0	9a		129.1
10		144.9	6${}^{\prime \prime }$	3.67 (m)	62.7	10		145.0
10a		122.6				10a		122.7

## Supplementary Materials

Supplementary materials, including HRMS, 1D and 2D NMR spectra for compound **1**, and computational details for **2**, are available online.
