# Supplementary material for: MS/MS-Guided Isolation of Clarinoside, a New Anti-Inflammatory Pentalogin Derivative
Source: Molecules. 2018 May 22;23(5):1237. doi: 10.3390/molecules23051237 (PMC6100466; doi:10.3390/molecules23051237)
Supplement: Supplementary file 1 [file molecules-23-01237-s001.zip › molecules-292954-supplementary.docx]

Supporting Information to MS/MS-Guided Isolation of Clarinoside, a New Anti-Inflammatory Pentalogin Derivative

Coralie Audoin^1^ , Adam Zampalégré^1^ , Natacha Blanchet^1^ , Alexandre Giuliani^2,3^, Emmanuel Roulland^4^ , Olivier Laprévote^4,5^ and Grégory Genta-Jouve^4^

^1^ Laboratoires Clarins, 5 rue Ampère, 95300 Pontoise, France;

^2^ DISCO Beamline, Synchrotron SOLEIL, 91192, Gif-sur-Yvette, France ;

^3^ UAR1008,CEPIA, INRA, 44316 Nantes, France ;

^4^ C-TAC, UMR 8638 CNRS, Université Paris Descartes, Sorbonne Paris Cité, Faculté de Pharmacie de Paris, 4 avenue de l’Observatoire, 75006 Paris, France;

^5^ Department of Biochemistry, Hôpital Européen Georges Pompidou, AH-HP, 75015 Paris, France

[Figure 1:](#_Toc509614578) ^[1](#_Toc509614578)^[H NMR spectrum of 1 in CD](#_Toc509614578)_[3](#_Toc509614578)_[OD (600 MHz). 2](#_Toc509614578)

[Figure 2: 13C NMR spectrum of 1 in CD](#_Toc509614579)_[3](#_Toc509614579)_[OD (125 MHz). 2](#_Toc509614579)

[Figure 3: COSY spectrum of](#_Toc509614580) **[1](#_Toc509614580)** [in CD](#_Toc509614580)_[3](#_Toc509614580)_[OD. 3](#_Toc509614580)

[Figure 4: TOCSY spectrum of](#_Toc509614581) **[1](#_Toc509614581)** [in CD](#_Toc509614581)_[3](#_Toc509614581)_[OD. 3](#_Toc509614581)

[Figure 5: HSQC spectrum of](#_Toc509614582) **[1](#_Toc509614582)** [in CD](#_Toc509614582)_[3](#_Toc509614582)_[OD. 4](#_Toc509614582)

[Figure 6: HMBC spectrum of](#_Toc509614583) **[1](#_Toc509614583)** [in CD](#_Toc509614583)_[3](#_Toc509614583)_[OD. 4](#_Toc509614583)

[Figure 7:](#_Toc509614584) ^[1](#_Toc509614584)^[H NMR spectrum of](#_Toc509614584) **[1](#_Toc509614584)** [after H](#_Toc509614584)_[2](#_Toc509614584)_[SO](#_Toc509614584)_[4](#_Toc509614584)_ [hydrolysis. . 5](#_Toc509614584)

[Figure 8:](#_Toc509614585) ^[1](#_Toc509614585)^[H NMR spectrum of 1 after H](#_Toc509614585)_[2](#_Toc509614585)_[SO](#_Toc509614585)_[4](#_Toc509614585)_ [hydrolysis (enlargement). 6](#_Toc509614585)

[Figure 9:](#_Toc509614586) ^[1](#_Toc509614586)^[H NMR spectrum of 1 after H](#_Toc509614586)_[2](#_Toc509614586)_[SO](#_Toc509614586)_[4](#_Toc509614586)_ [hydrolysis (enlargement). 6](#_Toc509614586)

[Figure 10:](#_Toc509614587) ^[1](#_Toc509614587)^[H NMR spectrum of 1 after H](#_Toc509614587)_[2](#_Toc509614587)_[SO](#_Toc509614587)_[4](#_Toc509614587)_ [hydrolysis (enlargement). 7](#_Toc509614587)

[Figure 11: HRMS/MS spectrum of](#_Toc509614588) **[1](#_Toc509614588)**[. 7](#_Toc509614588)

[Figure 12: FTIR spectrum of](#_Toc509614589) **[1](#_Toc509614589)**[. 8](#_Toc509614589)

[Figure 13: Energy profile of both C5-O and C10-O bonds. 8](#_Toc509614590)

Figure 1: ^1^H NMR spectrum of 1 in CD_3_OD (600 MHz).

Figure 2: 13C NMR spectrum of 1 in CD_3_OD (125 MHz).

Figure 3: COSY spectrum of **1** in CD_3_OD.

Figure 4: TOCSY spectrum of **1** in CD_3_OD.

Figure 5: HSQC spectrum of **1** in CD_3_OD.

Figure 6: HMBC spectrum of **1** in CD_3_OD.


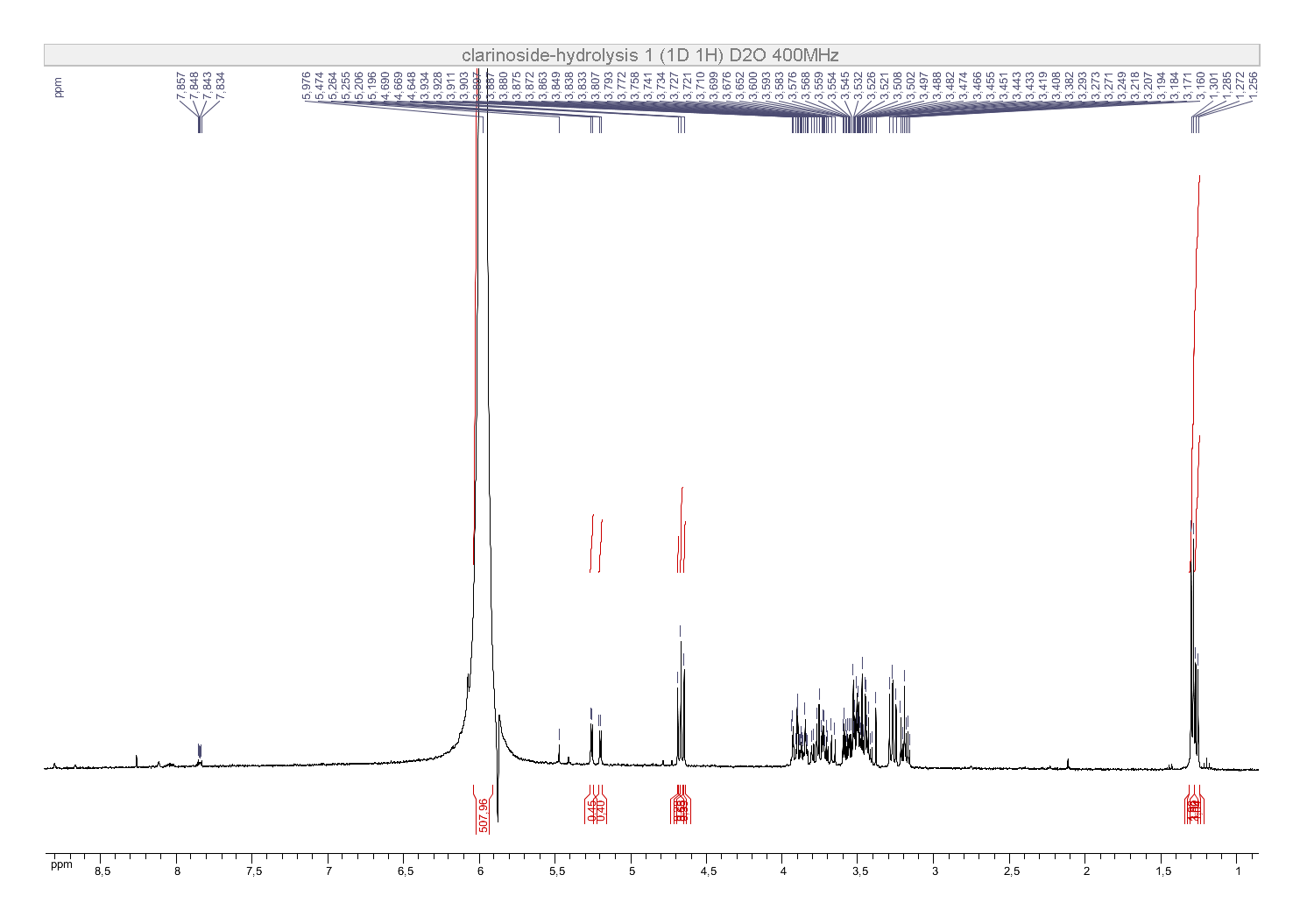


Figure 7: ^1^H NMR spectrum of **1** after H_2_SO_4_ hydrolysis.


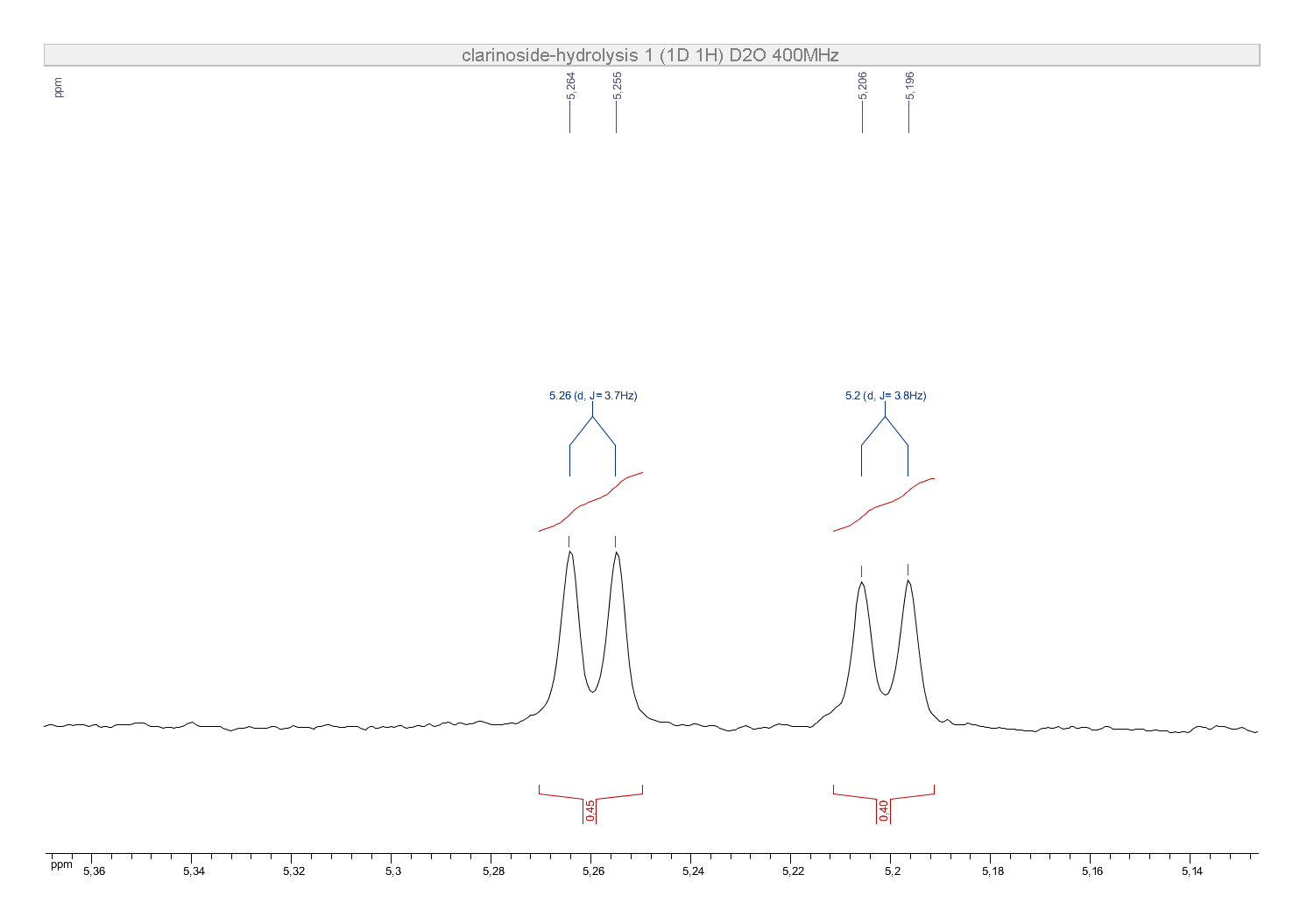


Figure 8: ^1^H NMR spectrum of 1 after H_2_SO_4_ hydrolysis (enlargement).


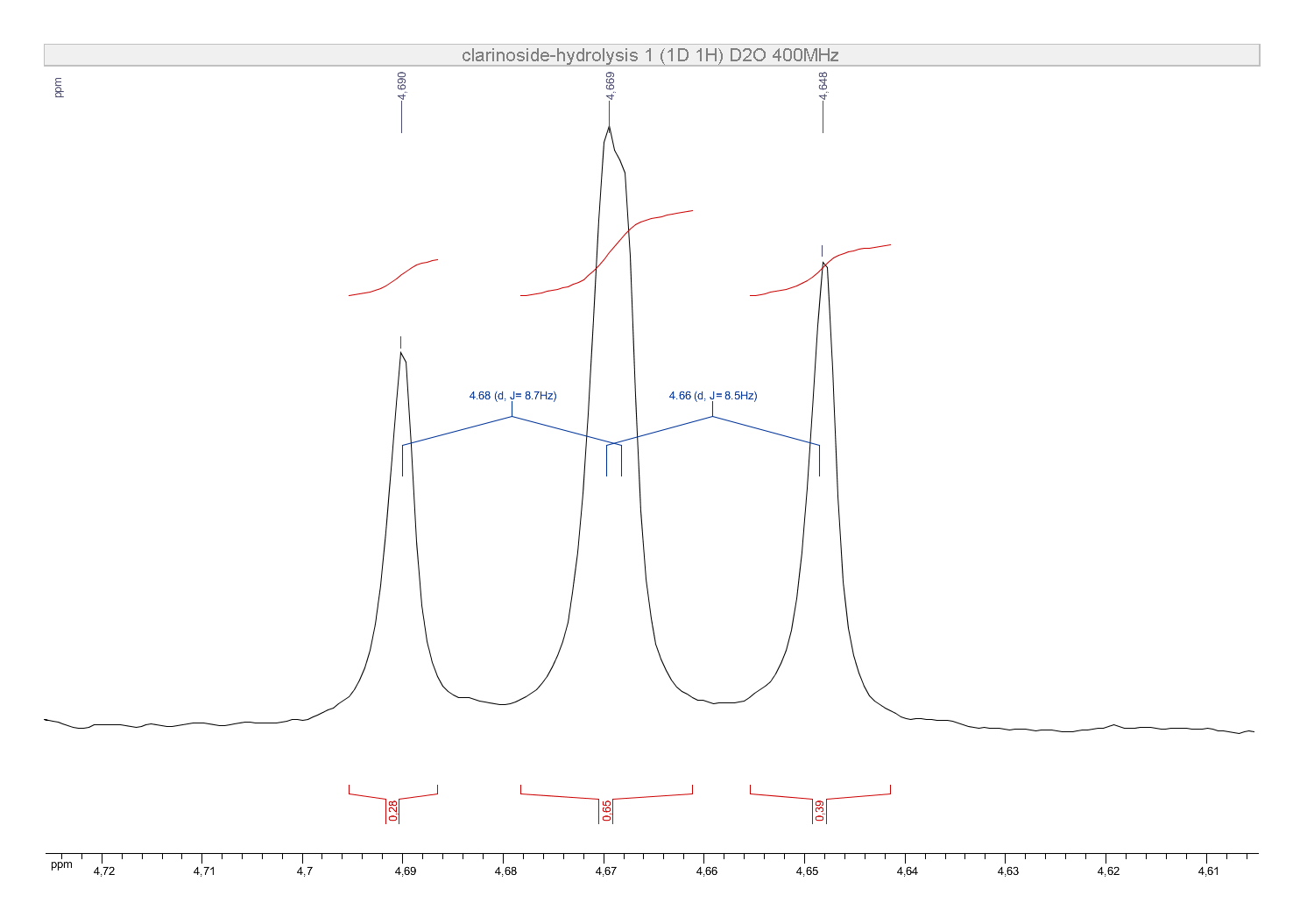


Figure 9: ^1^H NMR spectrum of 1 after H_2_SO_4_ hydrolysis (enlargement).


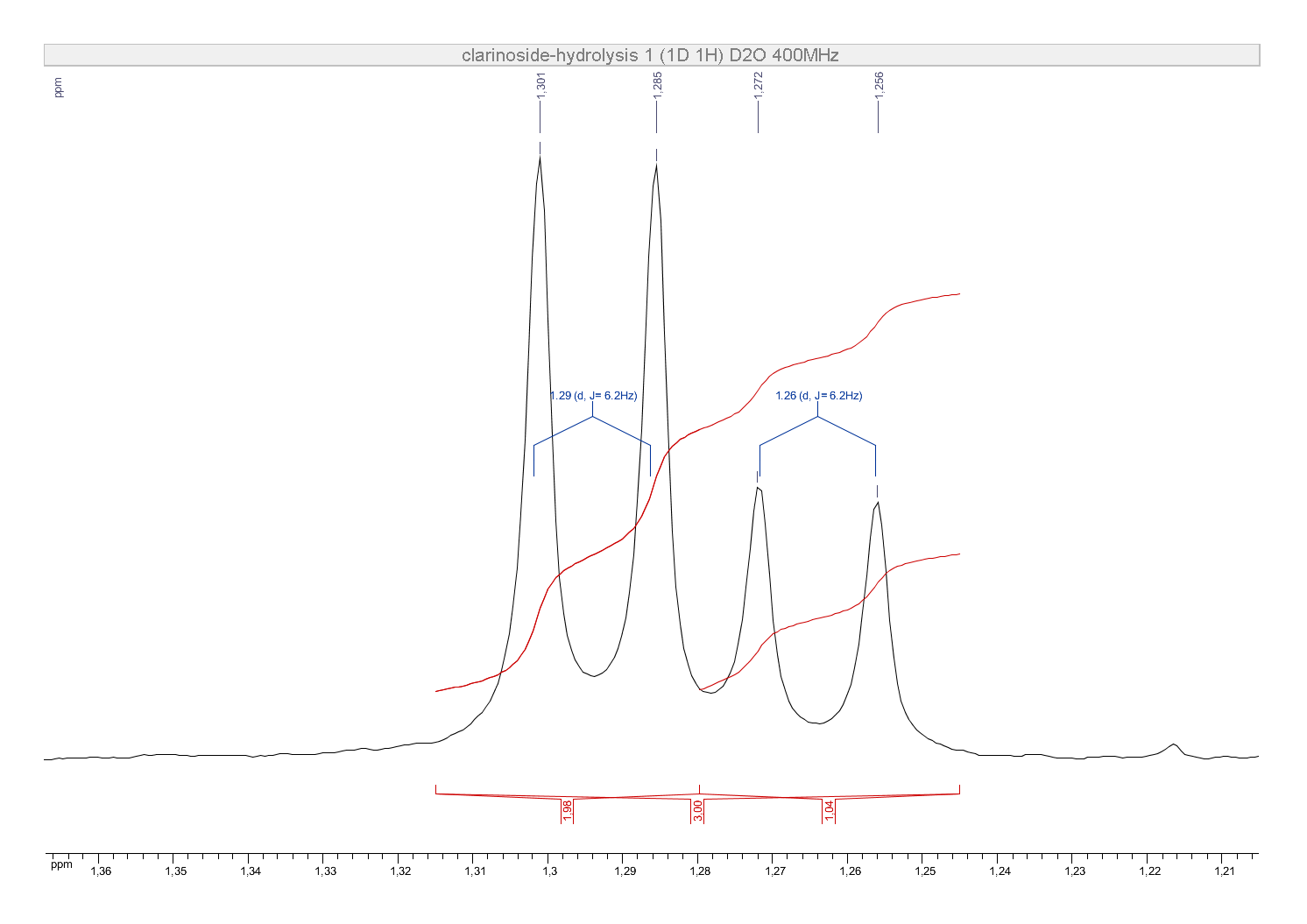


Figure 10: ^1^H NMR spectrum of 1 after H_2_SO_4_ hydrolysis (enlargement).


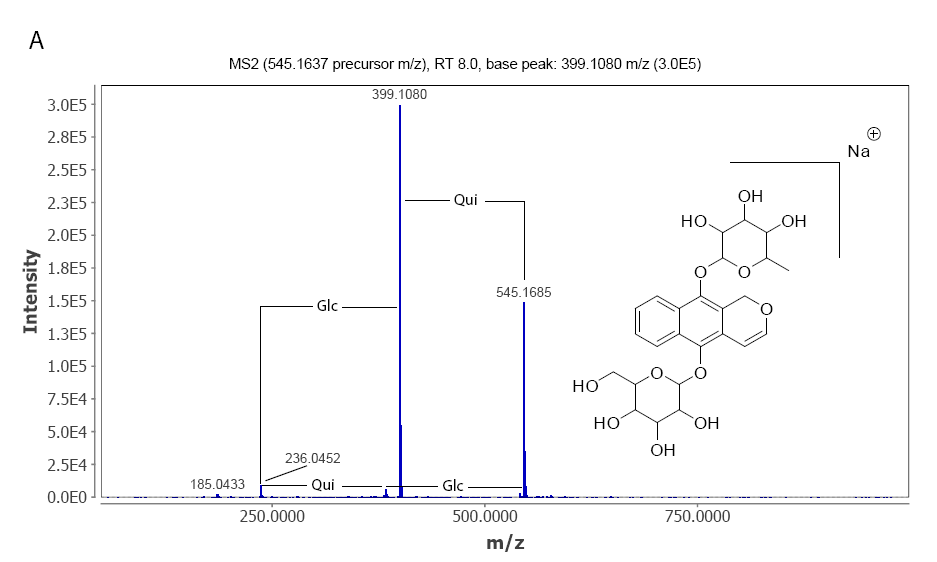


Figure 11: HRMS/MS spectrum of **1.**


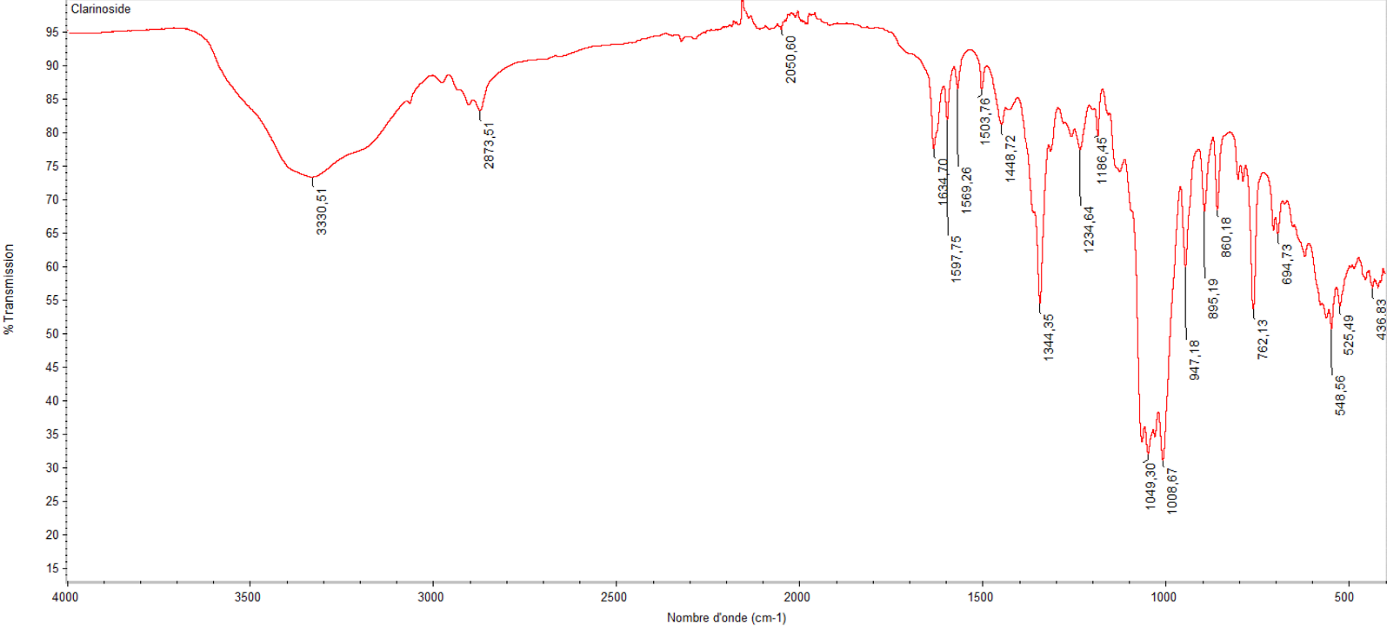


Figure 12 : FTIR spectrum of **1.**


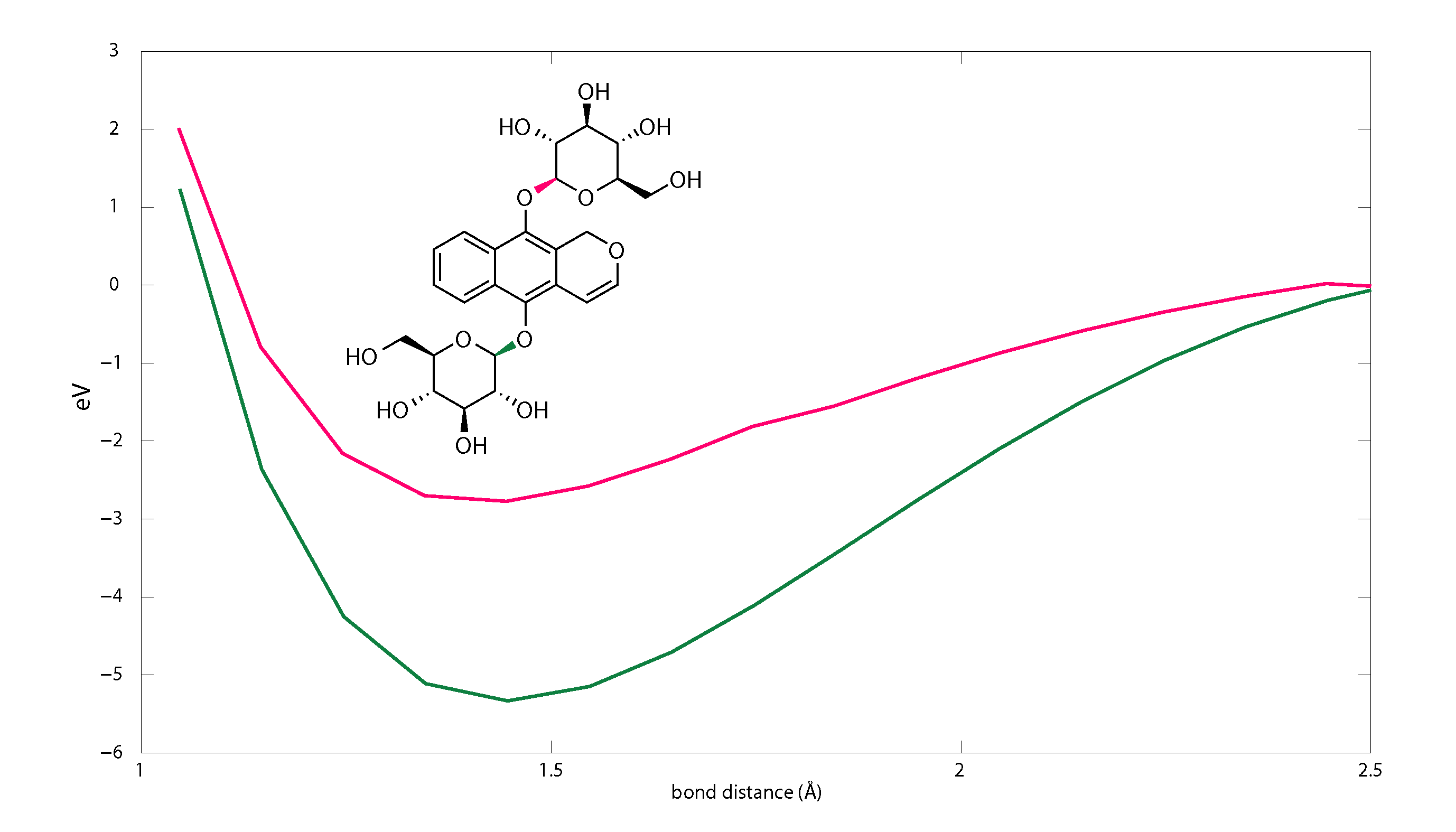


Figure 13: Energy profile of both C5–O and C10–O bonds.


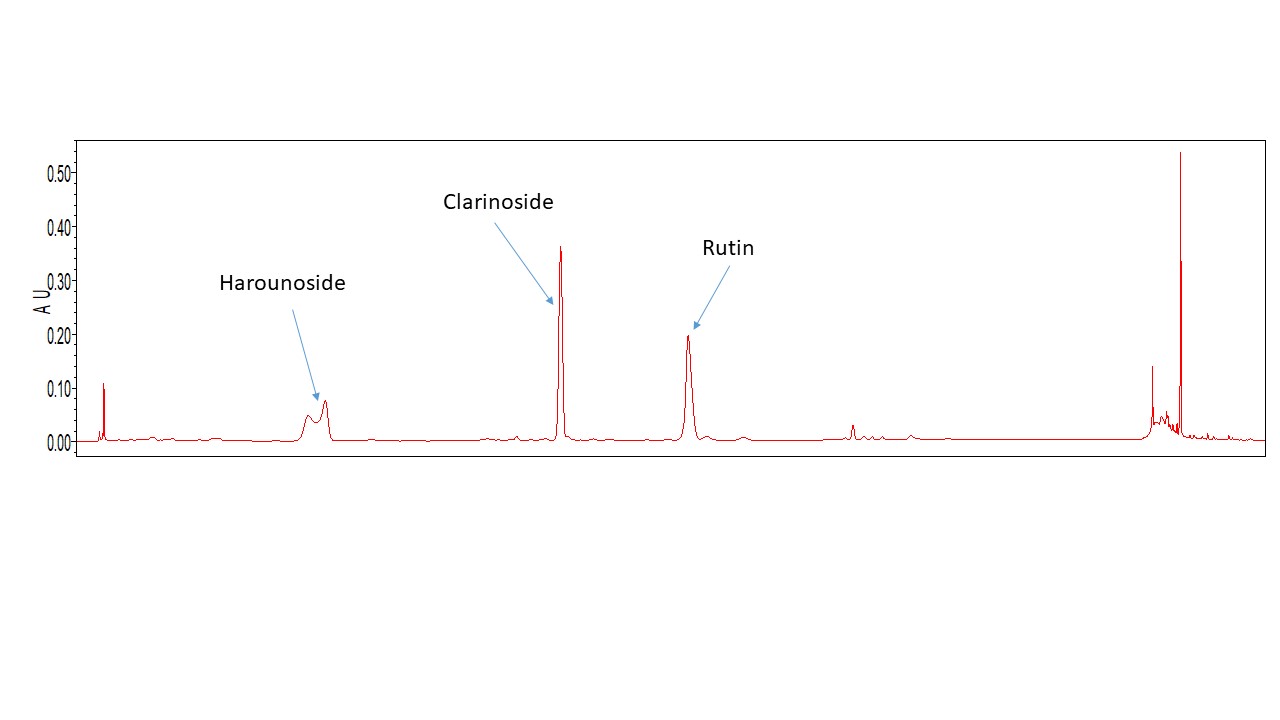


Figure 14: HPLC trace used for the separation (UV 254 nm).
